# Multifactor Quality and Safety Analysis of Semaglutide Products Sold by Online Sellers Without a Prescription: Market Surveillance, Content Analysis, and Product Purchase Evaluation Study

**DOI:** 10.2196/65440

**Published:** 2024-11-07

**Authors:** Amir Reza Ashraf, Tim Ken Mackey, Róbert György Vida, Győző Kulcsár, János Schmidt, Orsolya Balázs, Bálint Márk Domián, Jiawei Li, Ibolya Csákó, András Fittler

**Affiliations:** 1 Department of Pharmaceutics Faculty of Pharmacy University of Pécs Pécs Hungary; 2 Global Health Program Department of Anthropology University of California San Diego La Jolla, CA United States; 3 Department of Pharmaceutical Chemistry Faculty of Pharmacy University of Pécs Pécs Hungary; 4 Institute of Biochemistry and Medical Chemistry Medical School University of Pécs Pécs Hungary; 5 S-3 Research San Diego, CA United States; 6 Criminal Department Criminal Directorate Hungarian National Police Headquarters Budapest Hungary

**Keywords:** semaglutide, Ozempic, Wegovy, search engines, online pharmacies, patient safety, medication safety, nondelivery schemes, counterfeit, substandard and falsified medical products

## Abstract

**Background:**

Over the past 4 decades, obesity has escalated into a global epidemic, with its worldwide prevalence nearly tripling. Pharmacological treatments have evolved with the recent development of glucagon-like peptide 1 agonists, such as semaglutide. However, off-label use of drugs such as Ozempic for cosmetic weight loss has surged in popularity, raising concerns about potential misuse and the emergence of substandard and falsified products in the unregulated supply chain.

**Objective:**

This study aims to conduct a multifactor investigation of product quality and patient safety risks associated with the unregulated online sale of semaglutide by examining product availability and vendor characteristics and assessing product quality through test purchases.

**Methods:**

We used a complex risk and quality assessment methodology combining online market surveillance, search engine results page analysis, website content assessment, domain traffic analytics, conducting targeted product test purchases, visual quality inspection of product packaging, microbiological sterility and endotoxin contamination evaluation, and quantitative sample analysis using liquid chromatography coupled with mass spectrometry.

**Results:**

We collected and evaluated 1080 links from search engine results pages and identified 317 (29.35%) links belonging to online pharmacies, of which 183 (57.7%) led to legal pharmacies and 134 (42.3%) directed users to 59 unique illegal online pharmacy websites. Web traffic data for the period between July and September 2023 revealed that the top 30 domains directly or indirectly affiliated with illegal online pharmacies accumulated over 4.7 million visits. Test purchases were completed from 6 illegal online pharmacies with the highest number of links offering semaglutide products for sale without prescription at the lowest price range. Three injection vial purchases were delivered; none of the 3 Ozempic prefilled injection pens were received due to nondelivery e-commerce scams. All purchased vials were considered probable substandard and falsified products, as visual inspection indicated noncompliance in more than half (59%-63%) of the evaluated criteria. The semaglutide content of samples substantially exceeded labeled amounts by 28.56%-38.69%, although no peptide-like impurities were identified. The lyophilized peptide samples were devoid of viable microorganisms at the time of testing; however, endotoxin was detected in all samples with levels ranging between 2.1645 EU/mg and 8.9511 EU/mg. Furthermore, the measured semaglutide purity was significantly low, ranging between 7.7% and 14.37% and deviating from the 99% claimed on product labels by manufacturers.

**Conclusions:**

Glucagon-like peptide 1 agonist drugs promoted for weight loss, similar to erectile dysfunction medications more than 2 decades ago, are becoming the new blockbuster lifestyle medications for the illegal online pharmacy market. Protecting the pharmaceutical supply chain from substandard and falsified weight loss products and raising awareness regarding online medication safety must be a public health priority for regulators and technology platforms alike.

## Introduction

### Global Obesity Epidemic

Over the past 4 decades, obesity has escalated into a global epidemic, with its worldwide prevalence nearly tripling. In 2016, over 1.9 billion adults had a BMI between 25 kg/m^2^ and 29.9 kg/m^2^, classifying them as overweight, while over 650 million had a BMI of ≥30 kg/m^2^, falling into the obese category, accounting for 39% and 13% of the adult population, respectively [1]. If current trends continue, it is predicted that by 2035, more than half of the global population, exceeding 4 billion people, will be overweight, and 1 in 4 individuals, nearly 2 billion people, will be obese [2]. This alarming projection highlights the urgent need for the development of effective interventions, including new pharmacological treatments, given that overweight and obesity are key contributors to the global disease burden, ultimately leading to a decrease in life expectancy and quality of life [3-6].

Traditionally, weight loss management focuses on lifestyle modifications, diet, and exercise. However, these interventions alone are often inadequate for achieving and maintaining substantial weight reduction [7], prompting the development of surgical and pharmacological weight loss treatment options. The history of pharmacological weight loss treatments dates back over a century, with various medications being developed and subsequently withdrawn due to their low efficacy and severe adverse effects.

In the 1930s, amphetamines were introduced as a weight-loss treatment after their appetite-suppressing effects were discovered during narcolepsy studies [8]. However, their high abuse potential led pharmaceutical chemists to search for nonaddictive alternatives for appetite suppression. Around the same time, 2,4-dinitrophenol, a compound used in explosive manufacturing, was marketed as a weight loss drug and was subsequently banned by the United States Food and Drug Administration (FDA) due to its narrow therapeutic index and severe adverse effects [9,10]. Despite its ban from medical prescriptions, dinitrophenol has recently regained popularity among bodybuilders and extreme dieters seeking rapid weight loss, with several documented fatalities linked to dinitrophenol weight loss capsules obtained from illegal online vendors [11-14].

Noteworthy anti-obesity treatments developed in the 1990s, such as sibutramine and orlistat, faced their own challenges. Sibutramine was withdrawn from the market in 2010 due to increased cardiovascular adverse events, while orlistat’s labeling had to be revised to include a warning about the potential for severe liver injury [15]. Other anti-obesity drugs, such as Belviq (lorcaserin HCI), a controlled substance approved by the FDA and recalled from the US market in 2020, have also been detected as illegally sold drugs by online vendors even before they were approved for sale [16].

### Emergence of Glucagon-Like Peptide 1 Receptor Agonists and Concerns About Illegal Trade

In recent years, the landscape of weight-loss pharmacotherapy has evolved significantly, with the emergence of new incretin-based therapies targeting glucose and appetite regulation through glucose-dependent insulinotropic polypeptide and glucagon-like peptide 1 (GLP‑1) receptor agonists [17]. Although research into the long-term safety profile and possible adverse effects of these drugs is still evolving, current evidence supports their use as effective treatment options for managing overweight and obesity in individuals with and without diabetes [18]. Notable new incretin-based therapies include tirzepatide (Mounjaro and Zepbound), liraglutide (Victoza and Saxenda), and semaglutide marketed under the trade names Ozempic, Wegovy, and Rybelsus. Among these preparations, as of early 2024, only Zepbound, Saxenda, and Wegovy have been FDA-approved for chronic weight management in individuals who have at least 1 weight-related medical condition (eg, type 2 diabetes, hypertension, or high cholesterol). For instance, Ozempic is only approved for the management of type 2 diabetes, and its use by individuals without diabetes for weight loss is considered off-label use.

Despite a limited labeled indication, Ozempic has surged in mainstream popularity as an off-label cosmetic weight loss treatment due to widespread media attention and celebrity endorsements across various platforms [19]. This heightened popularity is reflected in a 2023 nationwide survey in the United States, with 22% of individuals reporting to have asked their health care providers about using Ozempic for weight loss, 15% have used it for weight loss themselves, and nearly half (47%) know someone who has used Ozempic [20]. However, this rapid rise in popularity has not been without concerns; 76% of medical practitioners worry about its potential misuse, 59% fear it will limit access for patients with diabetes, and 54% fear impending shortages [20].

The popularity of the off-label use of Ozempic for weight loss, paired with capacity limitations at several manufacturing sites, has resulted in ongoing shortages throughout the European Union and the United States [21-23] causing tangible difficulties for patients with diabetes and creating fertile conditions for the proliferation of illegal online pharmacies that aim to capitalize on the heightened demand to sell counterfeit or substandard versions of the medication. In fact, major regulators such as the FDA in the United States and the European Medicines Agency, as well as international organizations such as the World Health Organization (WHO) have issued statements warning about counterfeit versions of Ozempic.

### Study Objectives

In response to the rapidly evolving landscape of weight loss drug development, this study aimed to perform an in-depth multifactor investigation of the unregulated online trade of semaglutide through online pharmacy websites, examining online product availability and vendor characteristics, and assessing product quality through test purchases. This multiphase study expands on the results reported in a previously published short research letter by completely describing the methodology and characteristics of that study [24]. The aim of this study is to enable the development of effective strategies to combat this growing threat to patient safety, public health, and the integrity of the pharmaceutical supply chain.

## Methods

### Overview

**Figure 1:**
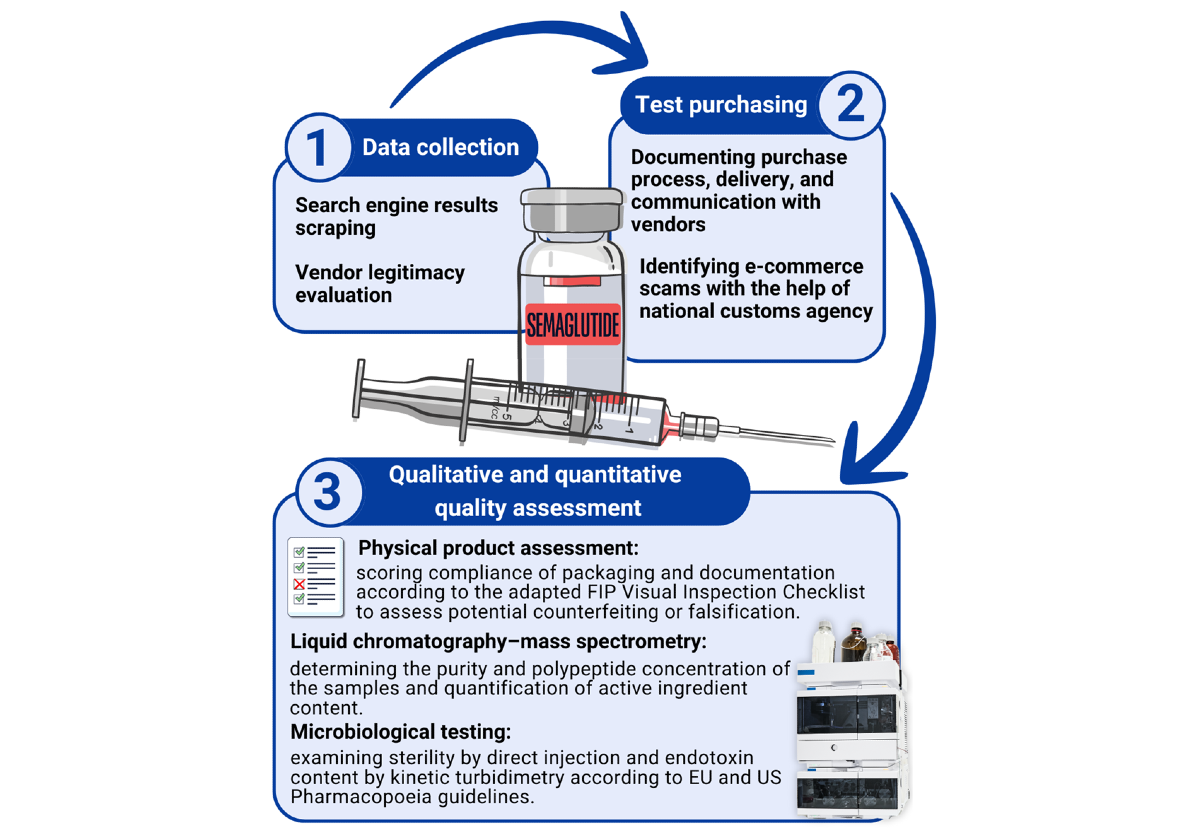
Summary of study methodology, including online market availability, website monitoring, packaging analysis, and complex product quality control testing. EU: European Union; FIP: International Pharmaceutical Federation.

This multiphase study was conducted in three phases: (1) manual and computer-assisted web market surveillance and content analysis for semaglutide products offered for sale online from July to September 2023; (2) completing a set of targeted test purchases from August to September 2023; and (3) completing a set of tests for pharmaceutical quality assessment, including examining logistics, visual package inspection, microbiological contamination, and chemical analysis of purchased products (Figure 1).

### Online Investigation and Search Engine Results Data Collection

In this first phase of the study, we aimed to identify the presence of illegal online sales of semaglutide, specifically from highly ranked search engine results of online pharmacies. We chose online pharmacies, rather than other potential sources of online sales (eg, social media advertisements, communication platforms, and dark web markets), as our unit of analysis because these sites are more conducive to content analysis related to patient safety concerns and pharmaceutical advertising and generally have e-commerce tools (eg, product menus and shopping carts) that facilitate test purchasing processes. We developed a market surveillance methodology, combining automated web crawling and search engine scraping with manual website evaluation similar to that used in prior published studies [25].

Google (Google LLC) and Bing (Microsoft Corporation) search engines were queried for “buy [Proprietary Name/API]” and “buy [Proprietary Name/API] without prescription” keyword combinations used to collect links from search engine results pages (SERPs) for Ozempic, Wegovy, and semaglutide. Data collection was performed using virtual machines hosted on Amazon Web Services and an automated web crawling program developed in Python (Python Software Foundation) using the Selenium package. Automated web crawling generated search engine results for both Google and Bing with all web browser cookie data, cached data, and search history deleted before conducting each search. To ensure that the links collected from SERPs were regionally relevant, geolocation and IP addresses were configured for study countries before data collection (Multimedia Appendix 1 [26-29]).

The following four categories were used to categorize the links: (1) legal internet pharmacies, (2) illegal vendors, (3) telemedicine websites, and (4) a collective *other* category for websites not providing pharmacy or telemedicine services. Websites were categorized as illegal pharmacies if the domain was classified as “rogue” by LegitScript (a private company that monitors and classifies online pharmacies based on their compliance with applicable laws and regulations and classifies them as “rogue” if they are operating out of compliance, illegally, or fraudulently) [30] or categorized as “not recommended” according to the National Association of Boards of Pharmacy (NABP) Safe Pharmacy verification database. NABP is a nonprofit organization that supports and works with the US state boards of pharmacy and maintains a list of websites that are reviewed for possible fraudulent and unsafe prescription medication sales online [31].

In addition, unverified pharmacy websites, whose domains were not listed in these databases, underwent a thorough manual evaluation to determine legitimacy. Two authors, ARA and AF, both licensed pharmacists, independently manually inspected every website, which was then followed by a joint discussion to reach a consensus on the legitimacy rating of all unverified websites not included in official databases. The manual website assessment was performed based on the following criteria: (1) indication of prescription requirement; (2) presence of sufficient patient guidance and product information; (3) presence of vendor’s address and location on the website; and (4) most importantly, whether a relevant and valid regulatory body certification, logo, seal of approval, or registration information could be found on the website. For the purpose of website content analysis and vendor selection, we selected only those domains that were featured >3 times in the aggregated search engine results.

### Vendor Selection Criteria and Test Purchasing

Websites included for content evaluation and test purchase were assessed based on the following variables: availability of formulation (prefilled injection pens or injection vial of lyophilized powder) of the parenteral semaglutide product, shipping information and geographical limitations, price, prescription requirement, comprehensiveness of product description (medication use, side effect information, promotion of off-label or unauthorized use, precautions, etc), vendor’s address and location, regulatory body logo, and seal of approval. We aimed to document potential medication and patient safety issues originating from information provided by online vendors, in line with methods used in our previously published studies that have examined aspects of safety risks associated with online pharmacies [32-35]. Websites inaccessible during our evaluation, referral sites (ie, affiliate sites) not offering the sale of products directly to customers, duplicate content websites (different domains with identical content), sites requiring submission of prescription documents before purchase, vendors not delivering products to Hungary or United States, and sites offering products above the previously defined price of US $200/single vial or pen were not included for detailed content analysis and test purchase. This price cutoff was chosen based on the average online price of semaglutide products that consumers would encounter at the time of the investigation, assuming they would opt for affordable and accessible choices when purchasing semaglutide products from online sources.

The overall rationale for this selection approach was to enable an exploratory study that assessed the highest-risk vendors based on previously known attributes (eg, low pricing and product availability) associated with illegal online sellers and the sourcing of substandard and falsified medicines. For the purpose of this study, we adopted the definitions and characterization of products as “substandard” or “falsified” consistent with the WHO definition and characterization of products as “counterfeit” consistent with both Hungarian and US regulations, given that these were the 2 destination countries for test purchases (further information on definitions is given in Multimedia Appendix 1). Each step of the online ordering procedure was photographed and simultaneously video recorded for future reference.

Credit cards, PayPal (PayPal Holdings, Inc), and bank transfers were the preferred payment options. Because chemical and microbiological analyses were to be performed in Hungary, the preferred shipping location was Hungary; however, if a seller did not offer direct delivery to this location, parcels were shipped to California, United States, and then forwarded to Hungary by express courier service.

### Assessment of Labeling and Packaging

The International Pharmaceutical Federation’s (FIP’s) checklist for visual inspection of medicines [36] was initially designed to aid health care workers in assessing whether a product is substandard or falsified. This evaluation encompasses a thorough assessment of the integrity of the container, which is essential to protect the product from external contamination and ensure its stability throughout its intended shelf life. The checklist also focuses on legal aspects, such as registration with the relevant drug regulatory authority and proper marketing authorization. Attention is given to the accuracy of information on labels, including the correct spelling of active ingredients and consistency between trade names and registrations. The checklist also addresses the authenticity of track and trace labeling, the explicit indication of storage conditions, and the inclusion of comprehensive informative leaflets. These elements are critical for maintaining consumer safety and ensuring compliance with applicable regulatory standards.

The authors have adapted the FIP checklist for the evaluation of packaging and labeling of delivered pharmaceutical products, based on the methodology outlined in the published work of Schiavetti et al [37], as well as our own preceding research [33]. This 22-item list is tailored to the specific dosage form of pharmaceutical products and accounts for significant advancements in protective measures that have been developed since the FIP list’s initial publication, such as serialization and implementation of data matrix or 2D codes on product packaging (Multimedia Appendix 1). The primary objective of this checklist was to rigorously evaluate the quality, safety, and regulatory compliance of medical products. The visual inspection involved observation and photo documentation of the primary and secondary packaging, leaflets, and labeling.

### Microbiological Testing

Sterility and microbiological contamination assessment tests were performed on lyophilized peptide samples obtained from sellers who successfully delivered purchased products to assess product quality and cleanliness of the manufacturing environment. Testing was performed at the International Organization for Standardization (ISO) 14644-1–certified microbiology laboratory of PharmaValid Ltd in Budapest, Hungary. Sterility testing was performed using direct injection technique according to European Pharmacopoeia (European Pharmacopoeia 11.0 2023 2.6.1) and United States Pharmacopeia (United States Pharmacopeia-National Formulary 2023 Issue 2, Chapter 71) guidelines, and bacterial endotoxin content measurement was performed via kinetic turbidimetry technique according to the European Pharmacopoeia (European Pharmacopoeia 11.0 2023 2.6.14) and US Pharmacopeia (US Pharmacopeia-National Formulary 2023 Issue 2, Chapter 85) guidelines. Both United States Pharmacopeia and European Pharmacopoeia standards were used to ensure that test-purchased product testing encompassed standards that applied to shipping destinations in both the United States and Hungary. Endosafe KTA2 Limulus Amebocyte Lysate reagent and *Escherichia coli* O55:B5 Control Standard Endotoxin were used. Samples were stored at 20 to 25 °C during analysis. In the case of 2 products, the content of each vial was dissolved in 2 ml of bacteriostatic water for injection provided by each vendor. One of the online vendors we purchased the product from did not provide the solvent necessary for reconstitution; therefore, endotoxin-free water was used during laboratory testing.

### Liquid Chromatography Mass Spectrometry Analysis

Stock solutions of the standard and the polypeptide samples were prepared in methanol. The working solutions were diluted using water/acetonitrile/formic acid (49/49/2, v/v/v). The estimated concentration of the polypeptide samples after dilution was 5 µg/mL. The final concentrations of the standard used for the calibration were 5, 1, 0.5, 0.25, and 0.1 µg/mL. To prevent polypeptide adsorption, every dilution step was carried out using low-protein binding Eppendorf microcentrifuge tubes. The prepared calibration standards and samples were transferred into 0.5 mL low protein-binding Eppendorf tubes, and 5 µL of each sample were injected from a special Eppendorf carrier plate. Chromatographic separation was performed using a Thermo Ultimate 3000 UHPLC system (Thermo Fisher Scientific) with a Luna Omega PS-C18 reversed-phase column (1.6 μm, 2.1 mm×150 mm inner diameter) from Phenomenex. For the multistep gradient-based separation method, we used 2 different solvents. Solvent A consisted of water/formic acid (99.9/0.1, v/v), while solvent B was composed of acetonitrile/formic acid (99.9/0.1, v/v). The gradient program included steps outlined in Multimedia Appendix 2. Data-dependent mass spectrometric acquisition was performed using a Bruker Maxis 4G UltraHigh Resolution Quadrupole Time of Flight instrument (Bruker Daltonics). The mass spectrometer was operated in positive ion mode, and the scanning range was set to 300 to 2200 m/z. The flow of nebulizer gas was 6 L/min at a pressure of 2 bar, and the temperature was set to 180 °C. The capillary voltage was 3.8 kV, and the 10 most intensive compounds were selected for collision-induced dissociation fragmentation. All data were processed using the Data Analysis software package (version 4.4, Bruker Daltonics).

### Ethical Considerations

Our study protocol was reviewed by the institutional review board at the University of Pécs (9710-PTE 2023) and was granted a waiver of ethics approval and consent as no human participants were participating in this study, and all information evaluated in our study was publicly available online and accessible via search engines and websites.

## Results

### Online Vendors Offering Semaglutide for Sale

Upon evaluating the 1080 links extracted from SERPs, we identified 317 (29.35%) links belonging to internet pharmacies, out of which 183 (57.7%) links directed users to legal pharmacies, and 134 links (42.3%) were identified directing users to 59 unique illegal online pharmacies and vendor websites. Notably, out of 59 illegal online pharmacies 21 (36%) were listed multiple times across the SERPs, with SemaSpace emerging as the most prevalent domain. In addition to parenteral products such as the Ozempic pen, we were able to identify offerings of unbranded injection vials of semaglutide and oral dosage form formulated tablets. Notably, oral formulations for semaglutide were not widely available through legal channels at the time market surveillance was conducted for this study.

Using the NABP’s Safe Pharmacy database to investigate the legitimacy of websites, we identified 47% (28/59) online pharmacies that were flagged as not recommended, whereas 53% (31/59) the remainder were not listed in the database, highlighting the challenge organizations such as NABP face in maintaining a comprehensive and current registry of illegal online pharmacies. Similarly, an evaluation through LegitScript’s database found that 47% (28/59) online pharmacies were classified as rogue, 24% (14/59) as unapproved, and 29% (17/59) as unlisted. Further emphasizing the elusive nature of these unregulated websites, it is important to highlight that 19% (11/59) of the domains were not listed in either database, which indicates that it is often challenging to identify and keep an up-to-date list of illegal online pharmacies, as these websites constantly cycle through being removed or shut down and new sites easily created and indexed.

A geographic analysis of the vendors revealed that nearly one-third (19/59, 32%) did not provide any address or contact information on their website, a clear indication of risk for counterfeit or illegitimate products. Among those disclosing their alleged locations, Canada and the United States were the most frequently listed, at 31% (18/59) and 22% (13/59), respectively. This was corroborated by WHOIS (a public directory service providing access to domain name registration information, eg, domain ownership and contact) registration data, which also indicated that most (26/59, 44%) of the domains were registered in these countries. Specifically, WHOIS registration data indicated that 31% (18/59) of the domains were registered to individuals based in the United States, 14% (8/59) were based in Canada, and 8% (5/59) were based in Iceland, while registrant location information for 24% (14/59) of the domains was withheld by the registrar, citing General Data Protection Regulation privacy laws as the reason. Investigating the hosting service provider data for the illegal online pharmacies revealed that a significant portion (45/59, 76%) of the websites were hosted by companies in the United States and Canada, with Cloudflare Inc being a preferred Canadian service provider, hosting more than a quarter (16/59, 27%) of the illegal websites detected.

Analysis of the web traffic data provided by SimilarWeb for the period between July and September 2023 revealed that the top 30 domains affiliated with illegal online pharmacies (Table 1) collectively accumulated an estimated 4.7 million visits, with a major share of traffic distributed among the top 5 websites, capturing more than 58% (2,730,848/4,705,502, 58.04%) of the total visits, indicating a substantial concentration of website traffic going to the top ranked websites indexed in structured search results. There was a considerable skew in visitor distribution, as toward the bottom of the top 30 list, the last 5 websites only accounted for a little over 1.55% (73,166/4,705,502) of the total visits, evidencing how website traffic drops for sites not prominently appearing in search engine results. The top sites included both conventional illegal pharmacies as well as newly emerging peptide-focused sellers, reflecting the increasing interest in purchasing peptide-based pharmaceuticals.

**Table 1:** Top 30 online vendor and intermediary website analytics and market surveillance data between July and September 2023.

Domain	LegitScript	Safe Pharmacy database (NABP^a^)	Location listed on the website	Domain registrant country (WHOIS)	Host	Server location	Total estimated visits between July and September 2023
muscleandbrawn.com	Not in the database	Not in the database	United States	Iceland	DigitalOcean LLC	United States	842,045
empowerpharmacy.com	Unapproved	Not in the database	United States	United States	Amazon Inc	United States	591,844
aminoasylum.shop	Rogue	Not in the database	—^b^	United States	Cloudflare, Inc	Canada	496,859
northwestpharmacy.com	Rogue	Not recommended	Canada	—	Cloudflare, Inc	Canada	447,587
canadapharmacy.com	Unapproved	Not recommended	Canada	United States	Amazon Inc	United States	352,513
canadadrugsdirect.com	Unapproved	Not recommended	Canada	United States	Amazon Inc	United States	215,126
corepeptides.com	Rogue	Not in the database	United States	Canada	Cloudflare London, LLC	United States	194,704
oddwayinternational.com	Rogue	Not in the database	India	India	Cloudflare, Inc	Canada	171,865
pharmaserve.com	Unapproved	Not recommended	Canada	—	Pretecs Networks Inc	Canada	143,664
insulinoutlet.com	Unapproved	Not recommended	Canada	United States	Google LLC	United States	132,143
biotechpeptides.com	Rogue	Not in the database	United States	Canada	Cloudflare London, LLC	United States	131,539
canadianpharmacyking.com	Rogue	Not recommended	Canada	Turks and Caicos Islands	Ntirety, Inc	United States	124,872
canshipmeds.com	Unapproved	Not recommended	Canada	—	Cloudflare, Inc	Canada	121,423
canadianinsulin.com	Unapproved	Not recommended	Canada	Canada	Cloudflare, Inc	Canada	101,742
israelpharm.com	Rogue	Not recommended	Israel	Israel	Cloudflare, Inc	Canada	93,549
semaspace.com	Not in the database	Not recommended	United States	United States	Cloudflare London, LLC	United States	75,686
buycanadianinsulin.com	Unapproved	Not recommended	Canada	Canada	Google LLC	United States	70,756
kiwidrug.com	Rogue	Not recommended	New Zealand	Panama	Solar Communications GmbH	Switzerland	56,360
oxygenpharm.cc	Not in the database	Not in the database	Canada	The Bahamas	Cloudflare Inc	Canada	48,222
polarbearmeds.com	Not in the database	Not recommended	Canada	Canada	BigScoots	United States	43,668
canpharm.com	Unapproved	Not recommended	Canada	—	Amazon Inc	United States	41,154
mydietdoc.com	Unapproved	Not in the database	United States	United States	Google LLC	United States	38,605
genx.bio	Not in the database	Not in the database	United States	Canada	Vultr Holdings LLC	United States	37,133
insulin.store	Not in the database	Not recommended	Canada	Iceland	Namecheap Inc	United States	32,185
australiaweightloss.com	Not in the database	Not in the database	Australia	—	Cloudflare, Inc	Canada	27,092
nevergiveupteam.com	Not in the database	Not in the database	Australia	United States	Cloudflare, Inc	Canada	21,358
help-pharm.com	Not in the database	Not in the database	—	Russia	Cloudflare, Inc	Canada	19,440
uschemlabs.com	Rogue	Not in the database	—	United States	Cloudflare, Inc	Canada	14,649
rxsavemeds.com	Rogue	Not in the database	United States	Malaysia	FiberState LLC	United States	9226
thebodyfollows.com	Unapproved	Not in the database	—	United States	Network Solutions LLC	United States	8493

^a^NABP: National Association of Boards of Pharmacy.

^b^Not applicable.

### Semaglutide Vendor Selection for Test Purchasing

A total of 6 online vendors offering parenteral semaglutide products were included in our content evaluation and test purchase phase (Table 2). To highlight the potential real-world issues of unregulated online semaglutide sales, we aimed to perform test purchases from what we consider higher-risk unapproved websites that were most likely visited by customers at the time of our evaluation. Consequently, these 6 rogue domains had the highest number of links (high prevalence) in all SERPs and offered semaglutide products for direct-to-consumer sale without prescription (easy access) at the lowest price range (affordability).

**Table 2:** Rogue online vendor characteristics and the semaglutide products purchased for testing in August 2023.

Vendor and product name	In the top 10 search results	LegitScript Verification	NABP^a^ category	Product form and dosage	Instructions (storage, administration, side effects, etc)	Communicated health-related benefits	Product price^b^ (+shipping fee)	Payment options	Shipped from (courier)
BiotechPeptides “Semaglutide (GLP‑1^c^) 3mg”	Yes	Rogue	Not recommended	Semaglutide vial (2 mg)	No	Appetite suppression and cardiovascular	US $113/vial (no shipping fee)	ACH^d^, CashApp, Venmo, and credit card	San Diego, California, United States (USPS^e^)
GeniusPharmacy “Ozempic- 0.25mg, 2Pens×1”	No	Rogue	Not recommended	Ozempic pen (0.25 mg)	Yes	Weight loss and diabetes	US $360/2 pens (+US $ 50)	Bitcoin and Zelle	Nondelivery scam (MailingOnTime)
PureMedsOnline “Ozempic Online - 0.25 mg 2 pens”	No	Rogue	Not recommended	Semaglutide vial (1 mg)	Yes	Diabetes, weight loss, and cardiovascular	US $300/2 pens (+US $30)	PayPal, Zelle, and bitcoin	Nondelivery scam (AsianaLogisticsExpress)
SemaSpace “Weeks 1-6 Bundle: 2 mg vial and bacteriostatic water 30 ml vial”	Yes	Not in the database	Not recommended	Semaglutide vial (3 mg)	Yes	Obesity	US $199/vial (+US $30)	PayPal only	New York, New York, United States (USPS)
USChemLabs “Semaglutide 1mg 5 Vials”	Yes	Rogue	Not in the database	Ozempic pen (0.25 mg)	No	Weight loss and blood sugar regulation	US $148.9/5 vials (+US $25)	Credit card, CashApp, and crypto currency	Camarillo, California, United States (FedEx)
WeightCrunchShop “Ozempic 0.25mg 1Pen”	Yes	Rogue	Not recommended	Ozempic pen (0.25 mg)	Yes	Weight loss	US $190/1 pen (+US $30)	Apple and Google pay, Zelle, bank transfer, Osko, and Bitcoin	Nondelivery scam (GLSCourierServices)

^a^NABP: National Association of Boards of Pharmacy.

^b^Smallest quantity offered for sale.

^c^GLP-1: glucagon-like peptide 1.

^d^ACH: payments through the Automated Clearing House network.

^e^USPS: United States Postal Service.

All included online vendors were categorized as illegitimate (eg, “not recommended,” “rogue,” or “unapproved”) by the LegitScript or NABP verification databases. In total, 3 (50%) of the 6 websites offered Ozempic injection pens for sale, while another 3 (50%) sold vials of lyophilized semaglutide powder to be reconstituted into a solution for injection. The price for the smallest dose and quantity of product available on the websites ranged from US $113 to US $360/purchase (mean $218.5, SD $93.6). One domain allowed only 1 payment method, while the rest offered numerous payment options, with 66% (4/6) vendors also offering cryptocurrency payments. None of the vendors requested prior medical prescriptions or any health-related information from patients before or during the purchase, and semaglutide pen sellers explicitly highlighted “no prescription” availability as marketing messages. All online vendors directly or indirectly referred to weight loss and obesity on the product page; thus, all the websites included in our study explicitly promoted unauthorized or off-label use of Ozempic or semaglutide-containing products. Instructions for use, storage, and administration were provided on 4 domains; however, 2 out of 3 lyophilized semaglutide peptide sellers did not communicate how to reconstitute, use, or administer the product.

The product descriptions provided by the lyophilized semaglutide peptide sellers presented their products ambiguously. On one hand, the ampules and the company logos suggested a research and laboratory focus, listing chemical properties, formulas, etc, with product labels indicating “research use only.” By contrast, most product descriptions provided by the peptide dealers emphasized the effects of GLP‑1 agonists, describing preclinical and clinical studies on potential health-related benefits (eg, beta cell protection, weight loss, diabetes, appetite reduction, and neurodegenerative disease). Both BiotechPeptides and USChemLabs listed numerous scientific publications and references to support the clinical benefit claims listed in their product descriptions.

**Figure 2:**
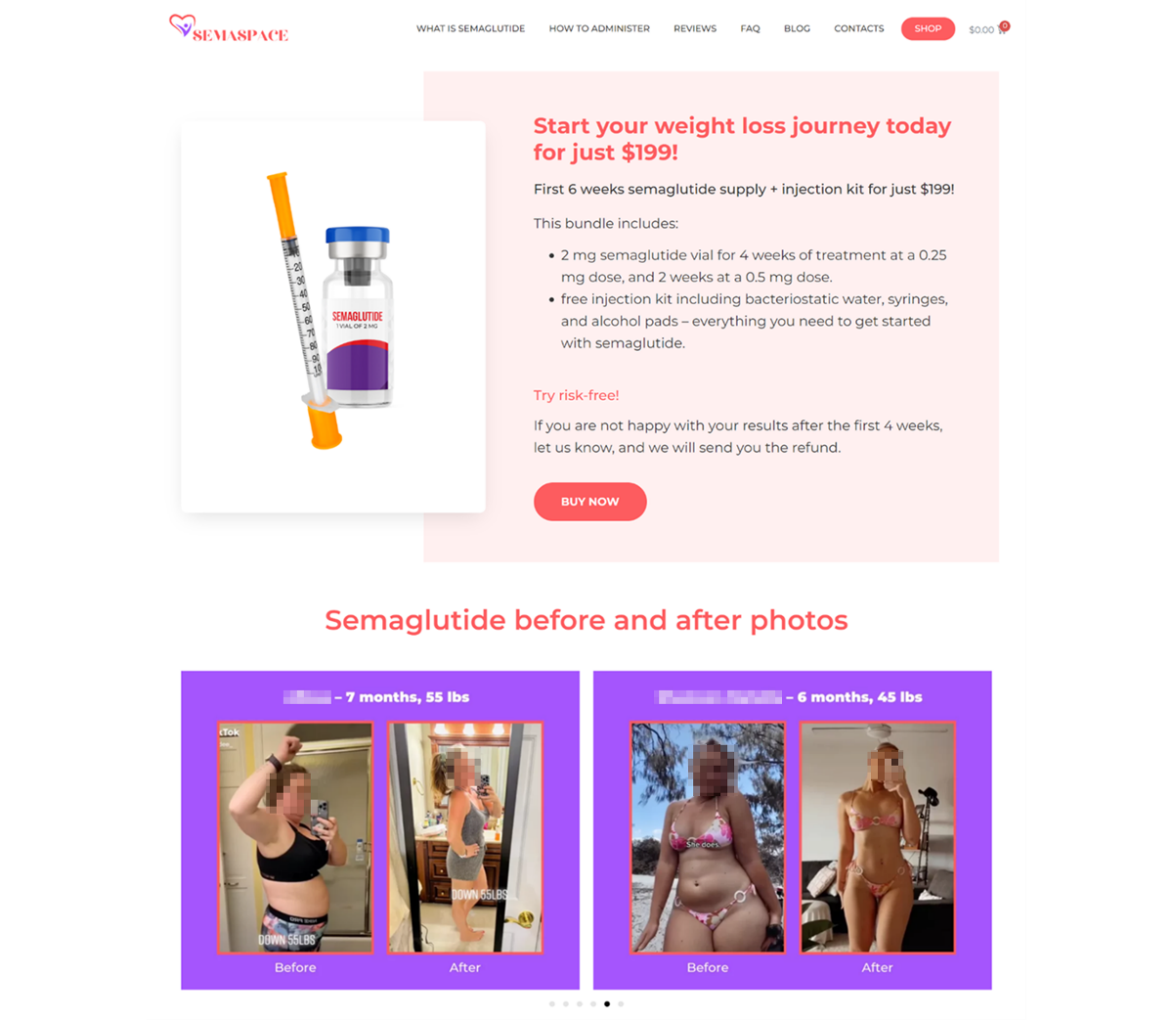
Screenshot of SemaSpace demonstrating common characteristics of illegal online pharmacies: off-label use promotion, no prescription requirements, unverified before-and-after photos to suggest efficacy, and misleading marketing strategies, such as risk-free trial and money-back guarantees. Personal identifiers have been blurred.

Regardless of the product formulation, all 6 websites permitted free access to semaglutide vials or prefilled injection pens without requiring any official documentation or professional information from the buyer. Thus, no prescriptions or patient health-related data were requested for Ozempic injections by the illegal vendors nor was any proof of personal qualification or corporate information required for research chemicals sold as injection vials by the peptide dealers. Although 2 lyophilized semaglutide peptide sellers seemingly discouraged human use of their “lab chemicals” and did not mention instructions on how to administer the product, the SemaSpace website, the other illegal pharmacy selling lyophilized semaglutide peptide, explicitly provided instructions on how to mix semaglutide with bacteriostatic water, along with syringes, alcohol prep pads, and dosing and injection guidance for customers (Figure 2). Prefilled Ozempic injection pen sellers listed information on product administration, precautions, and other pertinent information for consumer use. One website, WeightCrunchShop, contained numerous elements that are common among illegal internet pharmacy practice, including an emphasis on “without prescription” availability, “discrete delivery,” discount and “lowest price promise,” “money-back guarantee,” and related access to prescription-only products, such as ivermectin or ephedrine (Multimedia Appendix 1).

All online test purchases were completed within minutes from product selection to checkout. Most online vendors (4/6, 66%) offered untraceable payment methods. Furthermore, payment with cryptocurrency was encouraged and incentivized by offering a 5% discount or free shipping during the checkout process. On the BiotechPeptides website, customers had to sign the following statement before placing their order: “I agree to the terms and conditions. I am at least 21 years of age and understand that purchases are limited to licensed researchers and qualified professionals. I understand that the products listed on the site are not for human or animal use.*”* Similarly, the USChemLabs website’s terms and conditions also mention that “These products should only be used by Qualified Professionals. Bodily introduction into Human or Animals of any kind is strictly forbidden by law. All product information on this website is for educational purpose only.”

### Payment, Delivery, and Communication With Sellers

All purchases were confirmed by email. The automatic confirmation messages sent by the 3 Ozempic pen sellers were highly similar in layout and content and were created using the same open-source e-commerce platform (WooCommerce WordPress plugin). Only 2 (33%) of the 6 sellers (USChemLabs and BiotechPeptides) provided real-time credit card payment, while the rest (n=4, 66%) requested customers to complete their payment after receiving instructions by email following the online purchase.

Although purchases were confirmed via email, all further communication regarding payment and customs fees was initiated by sellers via WhatsApp Messenger (Meta Platforms, Inc). As cryptocurrency was the most preferred payment option, vendors provided guidance for customers on “How to pay with Bitcoin” using a credit card or cash app. However, because cryptocurrency payment is not available to all consumers and could be time consuming, we preferred and used more accepted payment solutions, such as PayPal or credit card. PayPal payments followed a similar process for all sellers. First, the vendors sent the PayPal username as an email associated with the vendor. Second, detailed instructions were sent by email, provided on the website, and communicated via WhatsApp on how to send the required payment amount. The payment instructions specified that the payment description field on PayPal could not contain anything except the customer’s name, and the payment type must be “For Friends and Family” (not for goods and services), or the order would be canceled and the money refunded, suggesting that vendors were requesting buyers to help conceal the nature of the payment.

**Figure 3:**
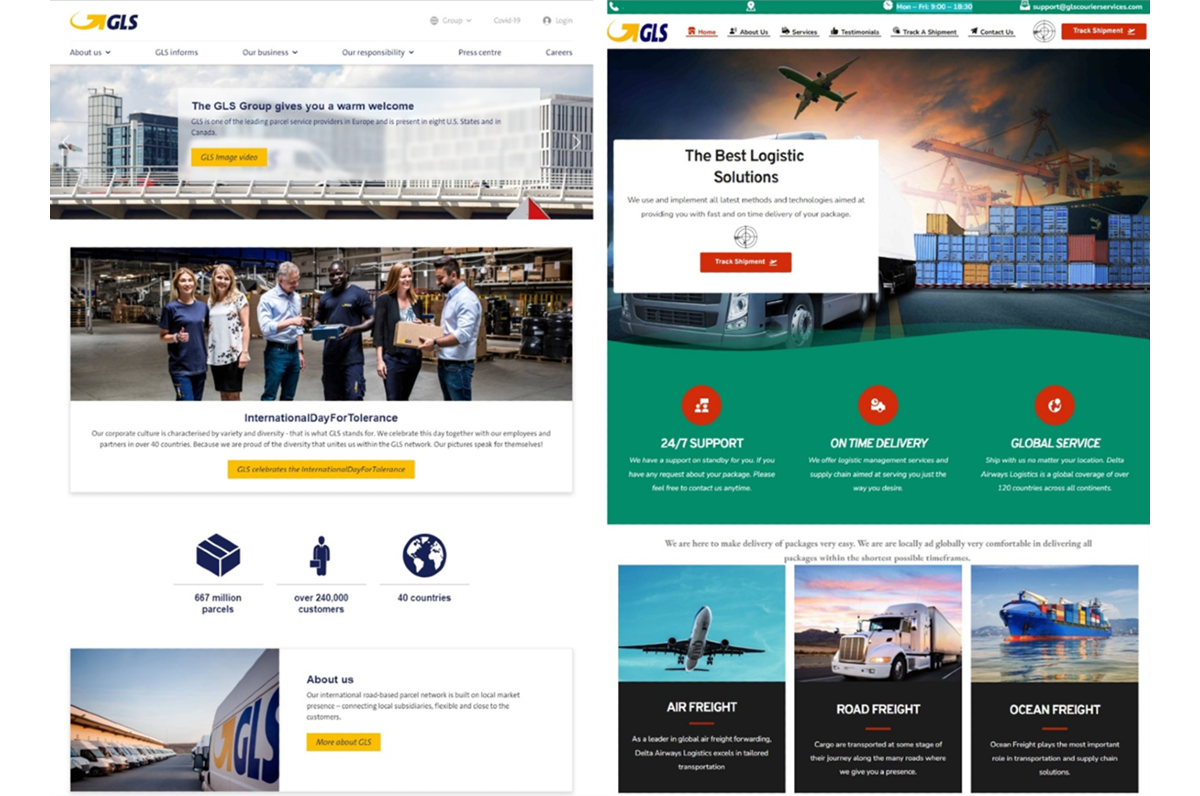
Screenshots of legitimate GLS Group on the left, and fraudulent GLSCourierServices website on the right. The legitimate website features authentic corporate branding, while the fraudulent website, which imitates the official GLS site, provides misleading information intended to deceive consumers. Note that GLS Group has since updated its logo, removing the yellow arrow [38].

All semaglutide injection vial products purchased were delivered; however, none of the Ozempic prefilled injection pens were received, as the illegal online pharmacies selling Ozempic pens used nondelivery e-commerce scams and the so-called customs clearance advance fee scam. After making the online purchases, vendors involved in nondelivery schemes referred us to third-party courier websites (AsianaLogisticsExpress, GLSCourierServices, and MailingOnTime) and provided fake tracking numbers that would show the packages were held up at customs. The sellers then demanded substantial sums of money to assist in releasing the purchased semaglutide medication, which they claimed was being held by local customs. These requested sums included US $1200 as an “insurance fee,” €450 (US $481.5) for “x-ray custom stamps,” and US $650 for “insurance and prescription stamps” to clear the supposedly stuck product through customs in Hungary. They assured us that the money would be refunded upon successful delivery. Upon investigation by the National Tax and Customs Administration, we confirmed that the packages listed as stuck in customs on the tracking website did not exist. Although the courier services, tracking codes, and delivery details could appear valid initially, they were entirely fraudulent and part of an elaborate e-commerce scam. For instance, GLSCourierServices clearly imitated the official GLS Group domain and misappropriated its logo to deceive consumers who fell victim to the e-commerce scam (Figure 3 [38]).

### Visual Inspection of Delivered Products

We adopted the FIP tool for visual inspection of medicines checklist for inspection of the delivered products, which we tailored to align with project-specific requirements, allowing us to evaluate semaglutide products against criteria essential for maintaining high standards of quality of a legitimate manufacturer (Table 3).

**Figure 4:**
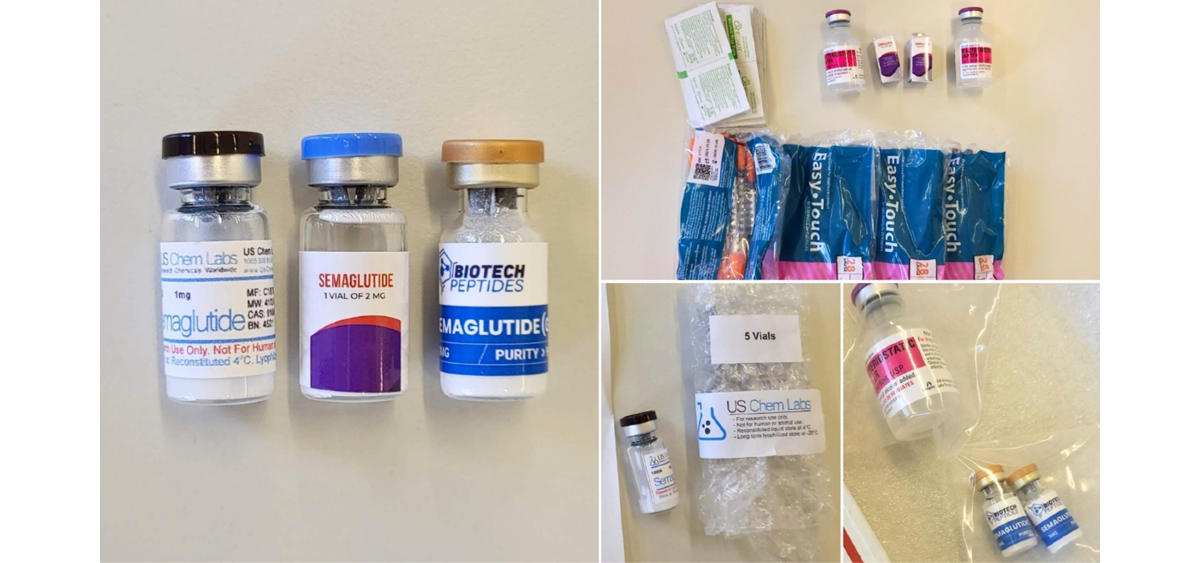
Injection vials containing lyophilized semaglutide peptide purchased from illegal internet pharmacies. USChemLabs and BiotechPeptides included purity information and warnings on the labels, while SemaSpace labeling did not. SemaSpace provided a complete injection kit with the semaglutide vials, BiotechPeptides provided bacteriostatic water with the semaglutide vials, while USChemLabs supplied semaglutide vials only.

Genuine Ozempic achieved a perfect score of 22 points, demonstrating full compliance with all assessed criteria. In contrast, products from SemaSpace, BiotechPeptides, and USChemLabs scored considerably lower, with 9, 8, and 8 points, respectively. The most notable discrepancies were observed in areas such as regulatory registration and oversight, accurate and legitimate labeling, and the provision of product-related information. Further visual inspection of the samples provided evidence that the products were unregistered or unlicensed. The FIP tool for visual inspection of medicines checklist revealed noncompliance in 13 (59%) and 14 (64%) out of 22 evaluated criteria, signifying a lack of compliance with quality, safety, and authenticity standards. Consequently, all test-purchased samples were classified as substandard, falsified, and counterfeit medical products (Figure 4).

**Table 3:** Results of visual inspection of products and corresponding scores for each semaglutide product.

Criterion	Genuine Ozempic pen	SemaSpace vial	BiotechPeptides vial	USChemLabs vial
Authentic track and trace labeling	Yes	No	No	No
Trade name legally registered	Yes	No	No	No
Trade name correctly spelled and includes ® symbol	Yes	No	No	No
Active ingredient name correctly spelled	Yes	Yes	Yes	Yes
Manufacturer’s name and logo legible and correct	Yes	Yes	Yes	Yes
Trade name and active ingredient match registered product	Yes	No	No	No
Product registered by manufacturer or agent	Yes	No	No	No
Dosage form registered and authorized for sale	Yes	No	No	No
Packaging and container protect from external environments	Yes	Yes	Yes	Yes
Container and closure appropriate for the product	Yes	Yes	Yes	Yes
Container safely and securely sealed	Yes	Yes	Yes	Yes
Container maintains product quality throughout shelf life	Yes	No	No	No
Carton and container labels match	Yes	Yes	No	No
Label information is legible and indelible	Yes	Yes	Yes	No
Manufacturer information and expiry dates indicated on label	Yes	No	Yes	No
Strength clearly stated on label	Yes	Yes	Yes	Yes
Dosage form clearly indicated on label	Yes	No	No	No
Dosage number clearly indicated on label	Yes	No	No	No
Labeled dosage form matches actual product	Yes	No	No	No
Manufacturer’s full address legible and correct	Yes	No	No	No
Storage conditions indicated on the label	Yes	Yes	No	Yes
Leaflet with dosage and use information included	Yes	No	No	Yes
Score and degree of compliance with the criteria (n=22), n (%)	22 (100)	9 (41)	8 (36)	8 (36)

### Microbiological Testing

Bacterial endotoxin content was measured using a kinetic turbidimetry assay at a detection limit of <0.01 EU/mL. Following sample dilution and the measurement of temporal turbidity changes, endotoxin levels for the USChemLabs and BiotechPeptides samples were found to be <2.8658 EU/mg and <2.1645 EU/mg, respectively. In the case of the sample obtained from SemaSpace, a more elevated endotoxin content of 8.9511 EU/mg was detected, indicating potential environmental contamination during production. The presence of endotoxin indicates residuals and remnants from the cell walls of Gram-negative bacteria. Despite the presence of endotoxin, all 3 lyophilized peptide samples were found to be devoid of viable microorganisms at the time of testing. The bacteriostatic water for injection supplied with the products was an off-the-shelf standard product and was not manufactured by the online vendor; therefore, the contaminants most likely originated from the water or nonsterile components used during the manufacturing of the lyophilized peptide. However, the exact source of the endotoxin cannot be determined.

### Quantitative and Qualitative Analysis

Semaglutide polypeptide can be identified and effectively quantified using mass spectrometry, as indicated by its characteristic peak at 1029.3 Da [M+4H]^4+^ in the mass spectrum, which serves as a critical reference point for evaluating the analytical measurements (Multimedia Appendix 2). The impurities found in products produced using automated peptide synthesis are highly variable. They can be unwanted by-products, solvent residues, or residues of chemicals used to protect the peptide chain. Due to their low molar mass and poor ionization capabilities, mass spectrometric identification of these substances can be challenging. To ensure the integrity of a pharmaceutical product, manufacturers must carefully monitor the manufacturing processes, which include monitoring the active pharmaceutical ingredient, impurities, and any potential contaminants that may arise from cross contamination from previous manufacturing processes. During our measurements, sample purity and quantity of the active ingredients were examined. The samples were tested for protein impurities; the chromatogram only displayed 1 signal for each delivered sample, indicating the exclusive presence of semaglutide (Multimedia Appendix 2). This indicates that the samples do not contain peptide-like impurities. However, a discrepancy emerged while comparing the purity levels provided by suppliers with the actual content of the active substance determined through our analysis. Documentation, available on vendors’ websites or shipped with the product, claims a purity of at least 99%. Contrarily, our analysis of products obtained from SemaSpace, USChemLabs, and BiotechPeptides revealed significantly lower polypeptide concentrations of 14.37%, 8.97%, and 7.7%, respectively. The quantity of semaglutide content in each sample also substantially exceeded the labeled amount, with excesses ranging from +28.56% to +38.69% (Table 4).

**Table 4:** Labeling accuracy and purity analysis of purchased lyophilized semaglutide samples compared with the original product obtained from the legitimate supply chain.

Sample	Total weight of powder in vial (mg)	Semaglutide content indicated on the label (mg)	Measured semaglutide content (mg), mean (SD)	Labeling accuracy	Purity indicated on label or website (%)	Measured purity (%), mean (SD)
Ozempic 1 mg solution for injection in prefilled pen (reference)^a^	—^b^	1	1.05 (0.02)	Semaglutide content exceeded the labeled amount by+5.05%	—	—
Biotech Peptides lyophilized semaglutide powder vial	50.1	3	3.86 (0.14)	Semaglutide content exceeded the labeled amount by+28.56%	99	7.70 (0.28)
SemaSpace lyophilized semaglutide powder vial	19.3	2	2.77 (0.12)	Semaglutide content exceeded the labeled amount by+38.69%	99	14.37 (0.63)
USChemLabs lyophilized semaglutide powder vial	14.9	1	1.34 (0.07)	Semaglutide content exceeded the labeled amount by+33.58%	99	8.97 (0.51)

^a^On the basis of the official Ozempic European public assessment report product information document published by the European Medicines Agency [39], 1 prefilled Ozempic 1 mg pen contains 4 mg semaglutide in a 3 mL solution. Pen is designed to deliver 4 doses of 1 mg (0.74 mL/dose).

^b^Not applicable.

These findings suggest the presence of low-mass, weakly ionizing impurities, although further studies using additional analytical techniques are necessary to conclusively identify contaminants. It is plausible that the observed impurities are an indication of the omission or incomplete execution of purification steps typically required at the end of the polypeptide manufacturing process, as they are time-consuming and costly, an indicative sign of substandard manufacturing practices used by unregulated or illegal online pharmacies [40,41]. The chemical analysis provides evidence for the online sale of out-of-specification (substandard) products along with falsified medical product classification based on the misleading labeling information.

## Discussion

### Principal Findings

Our study used a multifactor approach to assess risks associated with purchasing semaglutide online, identifying >300 links to online pharmacies, with close to half (n=134, 42.3%) directing users to 59 unique illegal online pharmacies, many of which sold semaglutide without requiring a prescription or any clinician oversight. On the basis of web analytics data, we estimate that the top 30 domains affiliated with these illegal online pharmacies accumulated over 4.7 million visits during the data collection phase of the study, between July and September 2023, a period when excitement and demand for semaglutide were accelerating [19,42]. Our test purchases from a selection of high-risk and easily accessible illegal online pharmacy websites revealed that half (3/6, 50%) of the transactions were fraudulent, with sellers engaging in e-commerce nondelivery scams, and the other half (3/6, 50%) of the transactions yielded clearly substandard and falsified products that were not manufactured to specifications and misrepresented their constituency based on labeled information. In fact, 2 of the websites evaluated by test purchases conducted in this study have subsequently been sent FDA warning letters for engaging in unlawful sales of unapproved and misbranded semaglutide [43,44].

Specifically, we identified 2 distinct online marketing approaches used by illegal online pharmacies for this medication, which is trending globally, commands high prices and is impacted by ongoing drug shortages. The first strategy involved e-commerce fraud coupled with customs clearance advance fee scams, also known as “nondelivery schemes.” These scams are a form of financial crime, where online actors set up fake internet pharmacy websites to entice buyers with uncontrolled access to prescription-only medications. The catch is that these illegal online pharmacies likely do not actually have any products in stock, which is why shortages do not affect them, and, of course, the purchased products are never shipped. Furthermore, these financial scams include referrals to fake third-party courier service providers that request additional fees to release packages from customs, an effort to scam the customers who fall for the original nondelivery scam a second time.

The second strategy involves selling dubious laboratory chemicals, such as lyophilized peptides shipped in glass vials, or fake generic products while promoting potential health benefits and off-label use. On the basis of the results of our test purchases, these vendors ship products as the purchased vials were delivered. However, through our extensive laboratory evaluation of these products, we determined that although they contained semaglutide, they were substandard and falsified products with considerably low purity levels and elevated presence of endotoxin sold through illegal online sales, corroborating findings of a recent NABP report and concerns raised by global regulators and public health organizations such as the WHO [45]. Furthermore, assay values that indicated the quantity of semaglutide content in tested samples substantially exceeded labeled amounts raise concerns about possible incorrect dosing or overdose of semaglutide, particularly when self-administering using the reconstituted product as purchased in this study. In fact, the FDA has issued similar warnings due to the detection of adverse events and hospitalizations associated with compounded semaglutide injections that were self-administered [46,47].

Opportunities for drug counterfeiting, diversion, financial fraud, and patient harm are likely to continue to grow as global demand for and widespread off-label use of semaglutide continue to be promoted. For example, social media posts by celebrities have facilitated the spread of misinformation [48-50] and hype news diffusion [51] related to the benefits of semaglutide. New technologies, including generative artificial intelligence and search engine chatbots, may also recommend illegal online pharmacies for purchasing semaglutide, as recently reported in a separate study by Ashraf et al [52], further exacerbating potential consumer exposure and harm. The business model of rogue online pharmacies takes an opportunistic approach triggered by this increased demand; popularity driven by online content and drug promotion; and restricted access due to prescription requirements, shortages, or high prices. This “same old recipe” was previously used by illegal online pharmacies for sildenafil in the past 2 decades and more recently for ivermectin during the COVID-19 pandemic [26,53].

The drug supply chain has faced increasing challenges over the past 15 years, with multiple drug shortages affecting legitimate supply chains worldwide [54]. The shortages of antidiabetic and obesity drugs, which began in 2022, continue to be a problem 2 years later. We have entered an era where short viral videos and endorsements by celebrities about these products can have a long-lasting effect on consumers and the entire health care system and drug supply chain [55,56]. This new phenomenon should be investigated in depth from a public health and psychological perspective, and the results should be incorporated into the education of health care professionals and supply chain security tools to detect and interdict counterfeit versions of medications.

In our previous research, we drew attention to the potential risks faced by consumers searching for drug-related information about medicines in shortage, as we identified multiple illegal online vendors offering unavailable medicines. We hypothesized that there is no shortage in the illegal online market; however, we did not purchase medicines subject to a shortage at that time due to safety, ethical, and legal concerns in 2011, 2014, and 2016 [57-59]. In this study, we encountered a similar experience, as illegal internet vendors did not communicate that the medicines were out of stock. In parallel, by completing online purchases, we can strongly assume that an offline shortage coexists with online shortages, which increases the financial risk for patients as rogue online pharmacies engage in nondelivery schemes.

In our previous research, when our research group test purchased the growth hormone somatropine, we identified low amounts of the active ingredient and out-of-specification substandard medications due to inappropriate shipping conditions. In the case of semaglutide vials purchased in this study, analytical and microbiological testing revealed sterile, lyophilized products without extensive degradation. Peptide sellers offer active ingredients of popular prescription-only medicines, communicate the potential health-related benefits, and provide instructions for the reconstitution of freeze-dried powders. However, they simultaneously claim that their unbranded “laboratory products” are only for research purposes and are not suitable for human use. Consumers are likely to be misled and confused by quality and purity-related statements, as research-grade 99% purity and pharmaceutical-grade product quality are distinctly different.

Hence, complex instrumental analysis is required to investigate the quality of online-ordered medical products, including pharmaceutical technology tests; Raman spectroscopy to differentiate between genuine and falsified or substandard products; and qualitative, quantitative, and microbiological tests determined by pharmacopeias. As shown in the study by Ahmed et al [60], substandard and falsified medicines obtained online may pass pharmacopoeial requirements, and the active ingredient can be identified in the products. Due to the complexity and rise in the use of biotechnology medicines, illegal online pharmacies have also turned to these products. It must be emphasized that the quality of an online-ordered product is only one potential source of risk; the potential misuse and the incorrect use of prescription-only drug products without the supervision of a medical practitioner are also of great concern and a source of potential patient harm [60-62].

The seemingly unrestricted proliferation and access to illegal online pharmacies pose significant global public health risks, exacerbated by the public’s lack of knowledge on pharmaceutical quality assessment, the distinctions between off-label and labeled use, and the increasing trend of online purchases. To mitigate these risks, it is crucial to integrate training on health products and the unique characteristics of medicines into educational curricula and implement public awareness campaigns on the safe and effective procurement of medical products. Such interventions can help reduce the demand for medications outside the regulated supply chain. One potential tool is the development and dissemination of an international consumer version of the FIP checklist, which could guide consumers in evaluating the purchased medicines and understanding the associated risks. Moreover, there is a notable gap in research analyzing this complex phenomenon with few studies using multifactor methodologies, such as the evaluation of search trends, website metrics, and the health risks associated with products purchased online, as conducted in this study. This study underscores the urgent need for comprehensive strategies to educate consumers, improve content moderation among search engines and service providers, and better enforce regulations to safeguard public health against the dangers posed by illegal online pharmacies and rogue marketplaces of medical products [40,41,62].

### Limitations

Comprehensiveness is a major strength and simultaneously a limitation of our study. Incorporation of market surveillance approaches (search engine scraping and website content analysis) and pharmaceutical quality control (visual inspection, microbiological, and chemical analysis) provides a general overview of aspects and concerns associated with online pharmacies and falsified or counterfeit medical products. At the same time, each element of our research methodology could have been elaborated more extensively (eg, larger sample size, assessment of additional verification databases, and toxicological analyses) to provide additional details and identify specific safety and risk characteristics. Major limitations include a relatively small sample size of test purchases based on risk assessment due to the high costs and complexities of conducting test purchases, which limits the generalizability of our findings to all online pharmacies selling semaglutide illegally. While our search methodology has been proven effective in previous studies, not all illegal online pharmacy operations may have been captured in our analysis. The ≤US $200 per item price cutoff, while reflecting average online prices at the time of investigation, may have excluded higher-priced illicit semaglutide products. In addition, our analysis only included online pharmacy vendors shipping to the United States and Hungary, which may not be representative of the global market for illicit semaglutide that likely includes sellers using social media platforms, poorly regulated e-commerce sites, and notorious illegal markets such as dark web marketplaces. Therefore, our findings may not capture the full range of counterfeit semaglutide products available, although the identification across highly visited search engine results is still a serious concern. Lack of reproducibility another limitation as selected illegal online pharmacies may have different production runs or not be accessible online at all for future studies. Although the possibility that product quality might have been compromised during courier delivery could be a concern, this also reflects the real-world conditions consumers experience when purchasing these products from online vendors.

### Conclusions

GLP‑1 agonists represent an exciting new class of drugs with great potential to improve health outcomes for diabetes, weight loss management, and other health conditions, but are not without their own concerns regarding appropriateness for use, safety, and now quality and accessibility. This study identified a significant presence of illegal online pharmacies selling semaglutide products from online searches, often without requiring prescriptions and promoting off-label use. These illicit vendors pose serious risks to consumer safety, as evidenced by the substandard quality of their products revealed through physical, chemical, and microbiological assessments. Moreover, the popularity of these illegal websites, attracting millions of visits, underscores the urgent need for enhanced regulatory strategies and consumer education. Illegal and unauthorized access to these drugs from an evolving array of online sources may exacerbate such concerns, particularly in the context of postmarket surveillance and identifying causes for an increasing number of adverse events being reported for semaglutide-containing products. Multifactor approaches, such as the ones pursued in this study, have the potential to better triangulate factors related to online risk while also helping to develop targeted interventions (eg, patient education, online content moderation, and law enforcement action against illegal online pharmacies) aimed at ensuring safe and appropriate use of GLP‑1s as they become more mainstream for patient care. Future research should continue to monitor consumer demand and online purchasing behavior, examine the role of emerging digital platforms and social media in the distribution of illegal pharmaceutical products, and propose solutions to improve the reliability and safety of search engine recommendations in the context of GLP-1s and online pharmacies.

## Appendix

Multimedia Appendix 1 Supplementary methods, supplementary additional results, adapted International Pharmaceutical Federation Checklist, additional study figures and examples.

Multimedia Appendix 2 Liquid chromatography–mass spectrometry analysis.

## Abbreviations

FDA: Food and Drug Administration
FIP: International Pharmaceutical Federation
GLP‑1: glucagon-like peptide 1
NABP: National Association of Boards of Pharmacy
SERP: search engine results page
WHO: World Health Organization

## Data Availability

The datasets generated during and analyzed during this study are available from the corresponding author on reasonable request.
