# Supplementary material for: Multifactor Quality and Safety Analysis of Semaglutide Products Sold by Online Sellers Without a Prescription: Market Surveillance, Content Analysis, and Product Purchase Evaluation Study
Source: J Med Internet Res. 2024 Nov 7;26:e65440. doi: 10.2196/65440 (PMC11582493; doi:10.2196/65440)
Supplement: Multimedia Appendix 1 [file jmir_v26i1e65440_app1.docx]

**Supplementary Materials**

**Supplementary Methods:**

***Online investigation and search engine results data collection***

Results of returned hyperlinks were then generated in JSON format, which was then exported into .xlsx format for manual review and annotation. The first 30 results for each keyword combination were recorded and examined manually, to allocate website categories for each result. The rationale for this approach is that most individuals using search engines click on links suggested on the first SERPs during their session, with the first 10 links receiving the majority of the organic user click rate. The calculated cumulative click-through rates after the first 30 results are generally viewed as negligible [1].

**Vendor selection criteria and test purchasing**

Each step of the online ordering procedure was photographed and simultaneously video recorded for future reference. A private email account for a virtual patient (age: 38, gender: female) was created. To simulate the patient's starting therapy regimen, two ampules/pens of 0.25 mg/dose equivalent product were ordered from each vendor that met the selection criteria outlined above, since the recommended dosing schedule for Ozempic provided by the manufacturer is 0.25 mg per week. The date of the online purchase, payment method, shipping method and fees, order and tracking number, all communication with sellers, information provided by courier services, customs procedures, and time of delivery were documented.

***“Substandard”, “Falsified”, and “Counterfeit” Medicines Definitions***

Definitions related to counterfeit medicines have evolved over the past four decades. Although the legal implications of each definition are somewhat different and national regulations are not harmonized, medical products (which include medicines, excipients, active substances, medical devices, their parts and materials) affected by counterfeiting can be classified into three, often overlapping, categories in the context of the WHO’s Global Surveillance and Monitoring System. Substandard or “out of specification” are those authorized medical products that fail to meet either their quality standards or specifications. Unregistered/unlicensed medical products are products that have not undergone evaluation and/or approval by the national or regional regulatory authority for the market in which they are intended to be marketed/distributed or used. Finally, falsified medical products deliberately/fraudulently misrepresent their identity, composition, or source.

Given the international variance in regulations, it is crucial to provide a legislative framework for the classification of counterfeit products, particularly when focusing on specific regions. For instance, the Hungarian national regulation is in line with the Council of Europe’s convention on the counterfeiting of medical products known as the MEDICRIME Convention [2], and “counterfeiting” is meant in its broadest sense of “falsification”.  Under Article 185/A of Act C of 2012 of the Criminal Code, medicine counterfeiting offenses constitute a health-endangering offense, meaning that any activity related to counterfeit medicines, such as the manufacture, distribution, import, export, transport through the country, is considered a criminal offense. In the United States, the regulation of counterfeit drugs is addressed under the Federal Food, Drug, and Cosmetic Act, specifically 21 U.S.C. § 331 which details prohibited acts related to adulterated, misbranded and counterfeit drugs, with penalties for violations outlined in 21 U.S.C. § 333 [3]. Additionally, the Food and Drug Administration Safety and Innovation Act of 2012 (FDASIA), often referred to as the “Counterfeit Drug Bill”, also includes provisions in Title VII Drug Supply Chain, which focuses on securing the pharmaceutical supply chain and preventing counterfeit drugs from entering the market [4].

**Supplementary Additional Results:**

*Results excluded from analysis:* Search engine results that were excluded from further analysis and were not internet pharmacies included links to informative websites, news and report websites and sites that did not directly engage in sale of pharmaceutical products (n/N%=615/1080=56.94%) and 148 links belonging to telemedicine provider websites (n/N%=148/1080=13.70%).

**Adapted International Pharmaceutical Federation (FIP) Checklist:**

|  | **Criteria** | **Verification** | **Check list** |
| --- | --- | --- | --- |
| 1 | Authentic track and trace labeling | Does the track and trace labeling (e.g., Data Matrix, QR code, hologram) look authentic and function correctly? | Yes No |
| 2 | Trade name legally registered | Is the medical product and trade name legally registered in the country by the Drug Regulatory Authority? | Yes No |
| 3 | Trade name correctly spelled and includes ® symbol | Is the trade (brand) name correctly spelled and does it include the ® symbol? | Yes No |
| 4 | Active ingredient name correctly spelled | Is the active ingredient name correctly spelled on the label? | Yes No |
| 5 | Manufacturer's name and logo legible and correct | Are the manufacturer's name and logo legible and correct? | Yes No |
| 6 | Trade name and active ingredient match registered product | Do the trade name and the active ingredient names correspond to the registered product? | Yes No |
| 7 | Product legally registered | Has the manufacturing company or its agent legally registered the product for sale in the country? | Yes No |
| 8 | Dosage form registered and authorized for sale | Is the medicine in this dosage form registered and authorized for sale in the country? | Yes No |
| 9 | Packaging and container protect from external environment | Does the packaging and container protect the product from external environmental factors (e.g., moisture, light)? | Yes No |
| 10 | Container and closure appropriate for the product | Are the container and closure appropriate and suitable for the product inside? | Yes No |
| 11 | Container safely and securely sealed | Is the container safely and securely sealed? | Yes No |
| 12 | Container maintains product quality throughout shelf life | Does the container and closure ensure that the product meets required specifications throughout its shelf life? | Yes No |
| 13 | Carton and container labels match | If a carton is protecting the container, do the labels on the carton and container match? | Yes No |
| 14 | Label information is legible and indelible | Is all the information on the label legible and indelible? | Yes No |
| 15 | Manufacture information and expiry dates indicated on label | Are the manufacture and expiry dates and related information (e.g., batch number) clearly indicated on the label? | Yes No |
| 16 | Strength clearly stated on label | Is the strength, the amount of active ingredient per unit, clearly stated on the label? | Yes No |
| 17 | Dosage form clearly indicated on label | Is the dosage form clearly indicated on the container label? (e.g., pre-filled injection pen) | Yes No |
| 18 | Dosage number clearly indicated on label | Is the number of dosage units per container clearly indicated on the label? | Yes No |
| 19 | Labeled dosage form matches actual product | Does the dosage form stated on the label match the actual dosage form of the medication? | Yes No |
| 20 | Manufacturer's full address legible and correct | Is the manufacturer's full address legible and correct? | Yes No |
| 21 | Storage conditions indicated on label | Are the storage conditions indicated on the label? | Yes No |
| 22 | Leaflet with dosage and usage information included | Does the packaging contain a leaflet explaining the dosage, medicine content, adverse effects, actions, and usage instructions? | Yes No |

**Additional Study Figures and Examples**

Examples of illegal online pharmacies offering semaglutide products for retail sale.

**Supplement 1. Figure 1.** Online pharmacy offering Ozempic without prescription displaying typical signs of illegal activity.


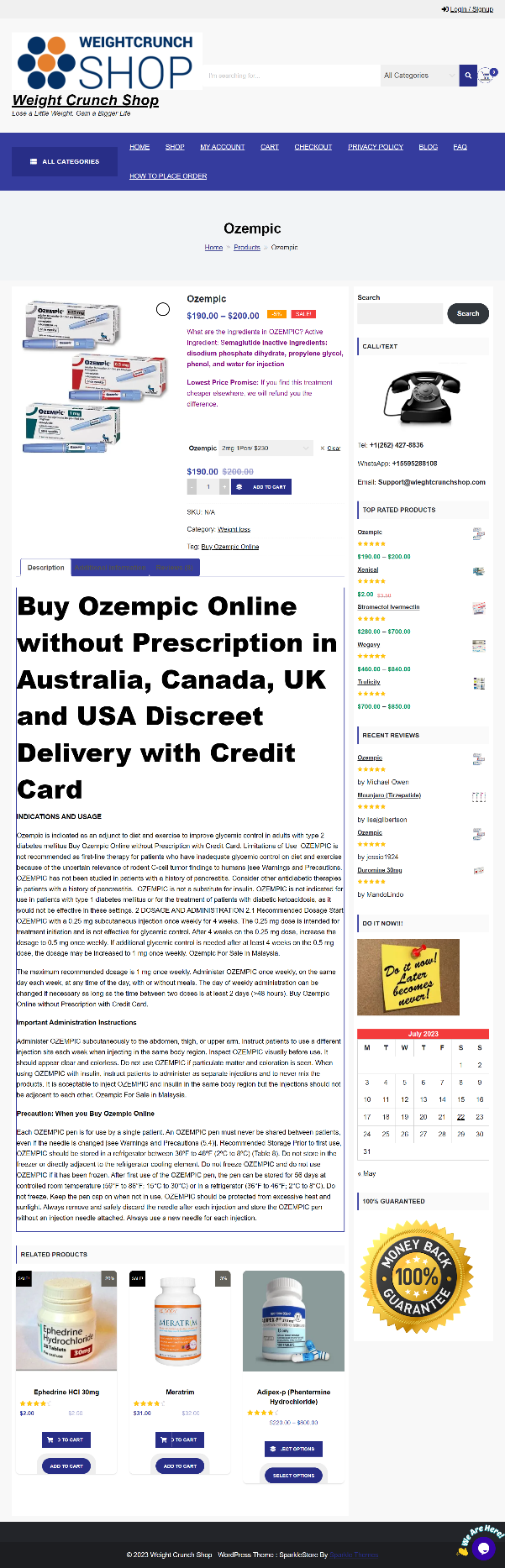


**Supplement 1. Figure 2.** Illegal online pharmacy advertising Wegovy with various dosage strengths, sold without prescription.


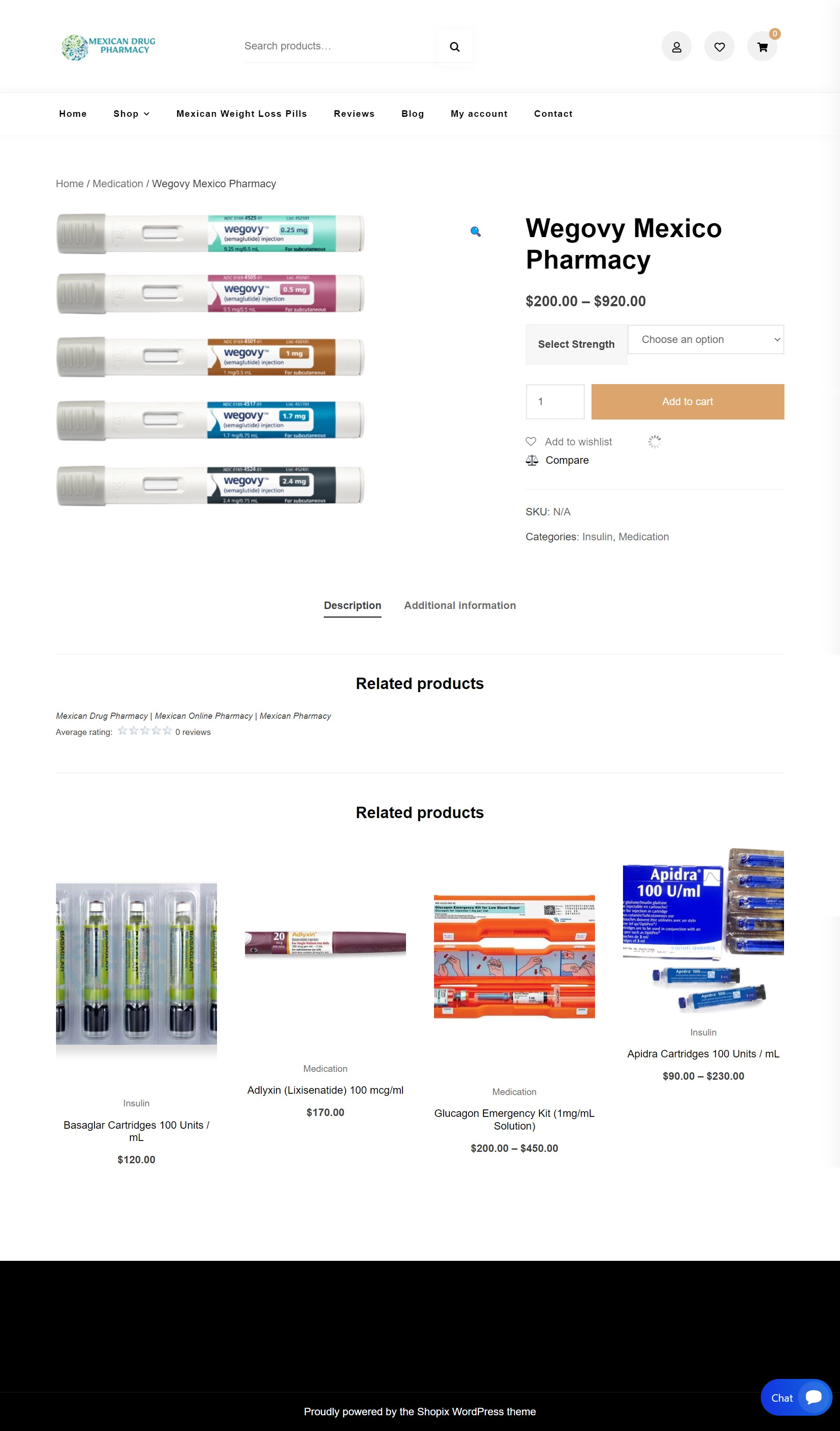


**Supplement 1. Figure 3.** Vendor marketing semaglutide (GLP-1) peptides for "research purposes only" sold without requiring valid prescription or documents related to research activities, offering bulk purchase discounts.


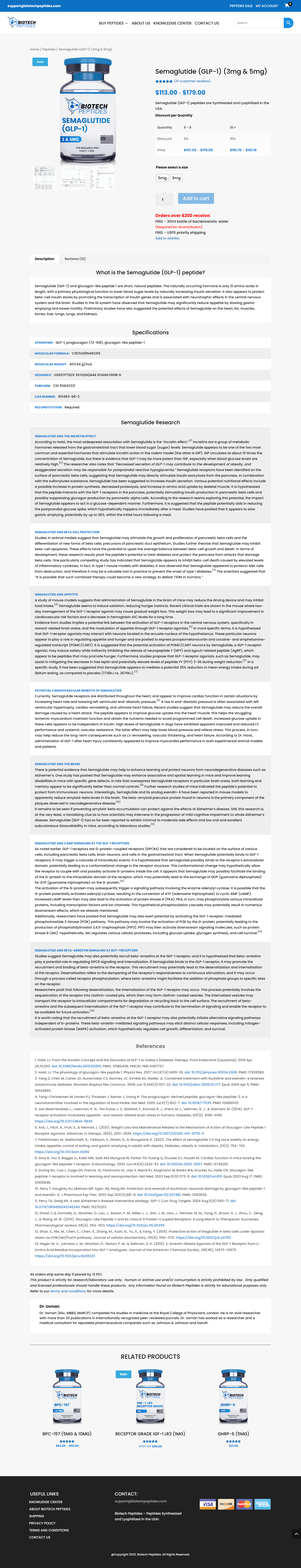


**Supplement 1. Figure 4.** Illegal online pharmacy promoting the sale of Wegovy without a doctor's prescription, featuring incentives such as discounts for cryptocurrency transactions.


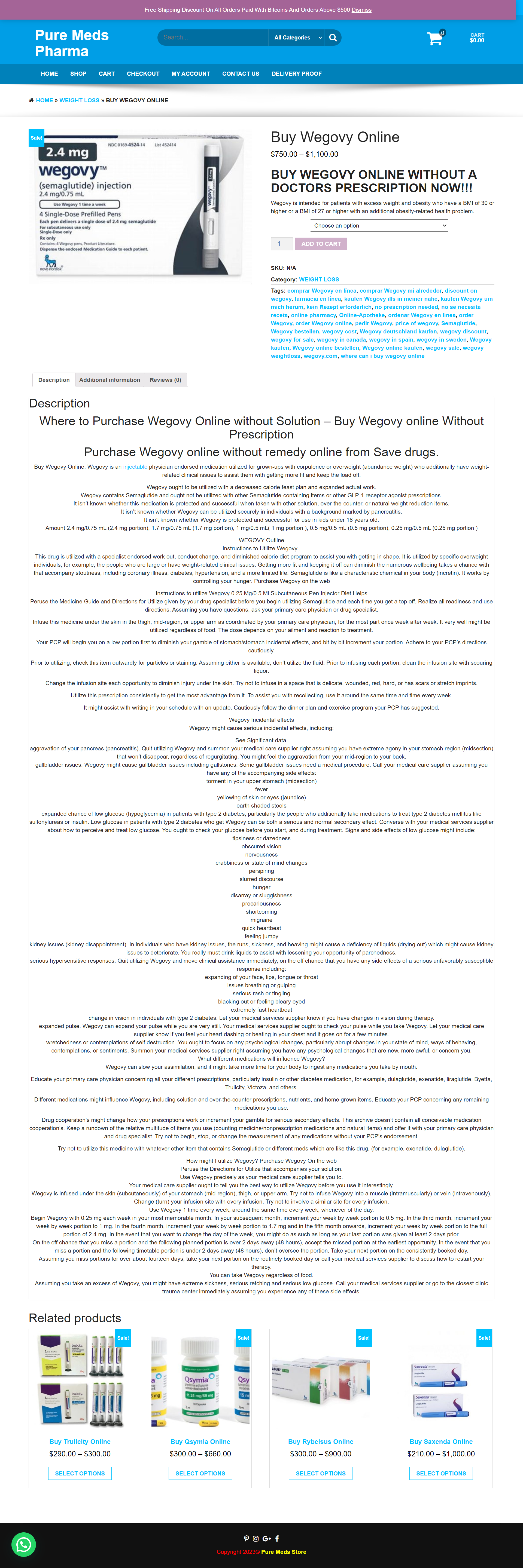


**References**

1. Fittler A, Paczolai P, Ashraf AR, Pourhashemi A, Iványi P. Prevalence of Poisoned Google Search Results of Erectile Dysfunction Medications Redirecting to Illegal Internet Pharmacies: Data Analysis Study. Journal of Medical Internet Research 2022 Nov 8;24(11):e38957. doi: 10.2196/38957

2. Council of Europe. Council of Europe Convention on the counterfeiting of medical products and similar crimes involving threats to public health (CETS No. 211). Council of Europe Treaty Office. Available from: https://www.coe.int/en/web/conventions/full-list [accessed Aug 12, 2024]

3. 21 USC CHAPTER 9, SUBCHAPTER III: PROHIBITED ACTS AND PENALTIES. Available from: https://uscode.house.gov/view.xhtml?req=granuleid%3AUSC-prelim-title21-chapter9-subchapter3&saved=%7CZ3JhbnVsZWlkOlVTQy1wcmVsaW0tdGl0bGUyMS1zZWN0aW9uMzMx%7C%7C%7C0%7Cfalse%7Cprelim&edition=prelim [accessed Aug 11, 2024]

4. FDA. FDASIA Title VII Drug Supply Chain Provisions. FDA FDA; 2019 Feb 9; Available from: https://www.fda.gov/regulatory-information/food-and-drug-administration-safety-and-innovation-act-fdasia/fdasia-title-vii-drug-supply-chain-provisions [accessed Aug 11, 2024]
