# Supplementary material for: Multifactor Quality and Safety Analysis of Semaglutide Products Sold by Online Sellers Without a Prescription: Market Surveillance, Content Analysis, and Product Purchase Evaluation Study
Source: J Med Internet Res. 2024 Nov 7;26:e65440. doi: 10.2196/65440 (PMC11582493; doi:10.2196/65440)
Supplement: Multimedia Appendix 2 [file jmir_v26i1e65440_app2.docx]

**Supplement 2. Liquid Chromatography Mass Spectrometry (LC‑MS) Analysis**

Gradient program included the following steps: 0.0-1.0 min, the composition was set to 0% B; 1.0-8.0 min, it was increased from 0% to 50.0% B; 8.0-10.0 min, from 50.0% to 100.0% B; 10.0-18.0 min the solvent composition was kept at 100% B; 18.0- 19.0 min, the composition decreased from 100.0% to 0% B, followed by a 6 min equilibration of the column. The flow rate was 200 µL/min.

**Supplement 2. Table 1**. Gradient conditions for semaglutide.

| Time (min) | 0.1% FA in DW (%) | 0.1% FA in ACN (%) | Flow rate (mL/min) |
| --- | --- | --- | --- |
| 0 | 100 | 0 | 0.2 |
| 1 | 100 | 0 | 0.2 |
| 8 | 50 | 50 | 0.2 |
| 10 | 0 | 100 | 0.2 |
| 18 | 0 | 100 | 0.2 |
| 19 | 100 | 0 | 0.2 |
| 25 | 100 | 0 | 0.2 |

**Supplement 2. Figure 1.** Mother and product ion spectra of protonated semaglutide ([M+4H]^4+^, m/z = 1029.3) by using Bruker Maxis 4G UHR-QTOF instrument.

**Supplement 2. Figure 2.** Chromatogram of the evaluated semaglutide samples by using Thermo Ultimate 3000 UHPLC™ system. The order of the samples is BiotechPeptides, SemaSpace and USChemLabs, respectively.

**Supplement 2. Figure 3.** Calibration line of the concentration determination.
